# Exploration of Core Microorganisms and Synthetic Microbial Communities in Low-Temperature *Daqu*

**DOI:** 10.3390/microorganisms13092044

**Published:** 2025-09-02

**Authors:** Panpan Chen, Dongsheng Zhang, Johane Johari Mkunga, Wenxi Zhai, Chunhui Shan, Xinquan Yang, Wenchao Cai

**Affiliations:** 1 Engineering Research Center of Storage and Processing of Xinjiang Characteristic Fruits and Vegetables, Ministry of Education, School of Food Science, Shihezi University, Shihezi 832000, China; 18154982958@163.com (P.C.);; 2 Key Laboratory of Processing and Quality and Safety Control of Specialty Agricultural Products (Co-Construction by Ministry and Province), Ministry of Agriculture and Rural Affairs, School of Food Science, Shihezi University, Shihezi 832000, China; 3 Office of the Party Committee of Xinjiang Production and Construction Corps, Urumqi 830002, China; 4 Dar-es-Salaam Institute of Technology, Dar-es-Salaam 11103, Tanzania

**Keywords:** low-temperature *Daqu*, core microorganisms, identification methods, synthetic microbial community

## Abstract

Light-flavor *Baijiu* (LFB) is renowned for its distinct flavor and long history, with the microbial community structure of low-temperature *Daqu* (LTD) serving as a crucial saccharification fermenter that significantly influences the quality and flavor of *Baijiu*. With the rapid advancement of biotechnology, research on LTD has become more in-depth, focusing on the identification of core microorganisms and the construction of Synthetic Microbial Communities (SynComs), which have emerged as research hotspots. Core microorganisms play a vital role in fermentation and flavor development, while SynComs are artificially constructed microbial combinations designed to optimize fermentation and improve liquor quality. This paper provides a systematic overview of the core microorganisms associated with LTD and their identification methods, as well as the concepts, advantages, applications, and construction methodologies of SynComs. It compiles relevant research findings to offer a theoretical foundation for a deeper understanding of the brewing mechanism and further optimization of the LFB brewing process, along with insights into future research directions.

## 1. Introduction

As one of the six major distilled spirits globally, Chinese *Baijiu* is not only a traditional alcoholic beverage with a rich history but also embodies profound cultural significance [1,2]. Its unique brewing processes and flavor characteristics are pivotal in the global distilled spirits industry [3]. Based on aromatic characteristics, *Baijiu* is categorized into three primary flavor types: strong-flavor, sauce-flavor, and light-flavor *Baijiu* [4,5]. Among these, light-flavor *Baijiu* (LFB) is celebrated for its “refined, crisp, and clean” style and has gained international recognition due to its distinctive production processes, including earthenware-jar solid-state fermentation, double distillation, and low-temperature malting, thereby positioning it as a key representative of Chinese *Baijiu* on the global stage [6,7].

**Figure 1:**
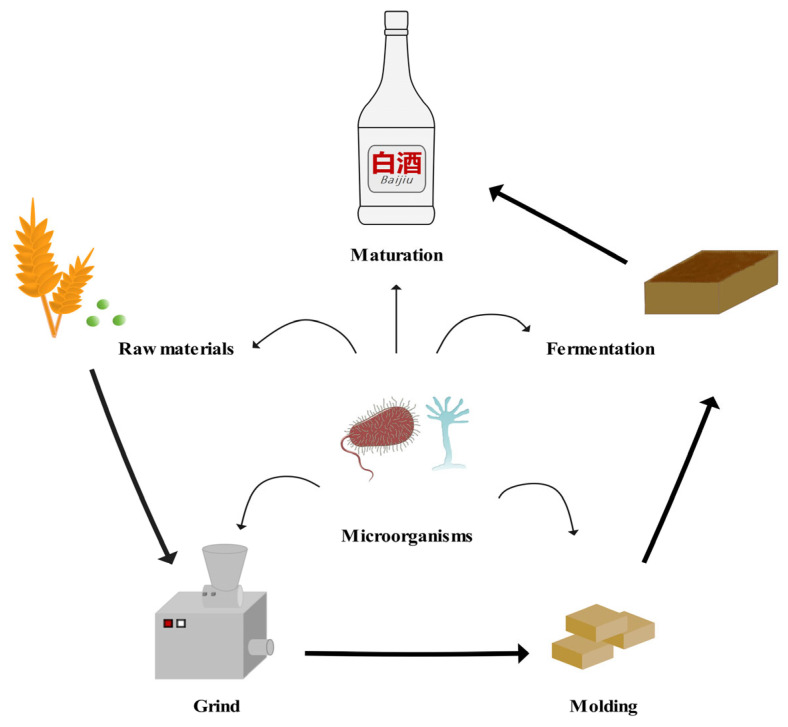
Manufacturing process of LTD.

The unique flavor and quality of LFB heavily depend on its proprietary saccharification and fermentation agent—low-temperature *Daqu* (LTD) [7,8]. As the core component of LFB production, LTD is crafted from ingredients such as wheat, barley, or peas, which are ground, shaped, and fermented for six months before use (Figure 1). This production process introduces environmental microorganisms through manual turning and temperature regulation, fostering a rich diversity of microbial life [9,10,11]. Unlike high-temperature and medium-temperature *Daqu*, LTD (fermentation peak temperature of 45–50 °C) imbues LFB with its characteristic sweet aroma, rich and mellow flavor, and clean aftertaste. The core of this process lies in the composition and activity of its microbial community [12]. In fact, the microbial community of LTD acts as the “invisible regulator” of LFB quality. Its fermentation involves a variety of microorganisms, including bacteria, yeasts, and molds, which produce enzymes and flavor compounds through metabolic activities, directly influencing the taste and aroma of LFB [13,14,15]. The stability of the microbial community is crucial for ensuring the consistency of LFB quality [16,17].

However, the production of LTD relies on natural inoculation in an open environment, where raw materials and microorganisms colonize without the addition of artificial fermentation agents [18]. This process leads to fluctuations in the microbial communities between different batches of LTD, resulting in inconsistent LFB quality, which poses a significant bottleneck to its industrial production and quality enhancement [19]. Addressing this bottleneck requires accurate analysis of the core functional microorganisms in LTD and the construction of stable Synthetic Microbial Communities (SynComs) [19,20]. The rapid development of molecular biology techniques has provided powerful tools for achieving this goal. Gene sequencing, polymerase chain reaction (PCR), and metagenomics have significantly advanced the identification of core microorganisms within LTD and the analysis of their functionalities, laying the groundwork for subsequent research [21,22,23]. Furthermore, SynComs, as an innovative solution for stabilizing traditional fermentation processes, demonstrate considerable potential across various fields, including food fermentation and agriculture [24,25,26,27,28,29]. In the context of LFB, previous studies have improved LTD flavor by introducing functional microorganisms like *Bacillus velezensis* and *Bacillus subtilis*, while also attempting to regulate the fermentation process through SynComs, which provides a theoretical foundation for constructing LTD SynComs [30].

Accordingly, this paper aims to introduce the research progress on core microorganisms and SynComs in LTD. It summarizes methods for identification and functional analysis of core microorganisms while describing the current application status and challenges of SynComs in LTD. By integrating interdisciplinary perspectives from synthetic biology and flavor science, this review not only offers theoretical support for achieving precise flavor customization and reinforcing the “refined and pure” style in LFB but also seeks to provide practical solutions for addressing quality stability issues in the industrial production of LFB, ultimately guiding related research and industrial applications.

## 2. Tapping into Core Microorganisms

### 2.1. Core Microbiota

#### 2.1.1. Based on Dominant Microbiota

Dominant microbiota refers to core microorganisms characterized by their abundance and frequency. However, the core microbiome is more rigorously defined as the set of microorganisms that are consistently associated with a particular environment across time (such as throughout fermentation) or space (such as across different production batches or seasons), and it is often linked to key functionalities [31,32,33]. During the brewing of LFB, the microbial community structure plays a crucial role in fermentation and flavor development. LTD constitutes 9.1% to 27.4% of the total bacterial community abundance and 61.1% to 80.0% of the total fungal community abundance during LFB fermentation, as quantified by high-throughput sequencing (HTS) technology [34,35]. Longitudinal studies tracking microbial succession have shown that while the relative abundances of these dominant groups shift significantly, key bacterial genera such as *Lactobacillus*, *Bacillus*, and *Acetobacter*, alongside fungal genera like *Aspergillus*, *Rhizopus*, and *Saccharomyces*, persist throughout the entire fermentation process, each dominating different stages [32]. The remaining microbial content may originate from non-dominant taxa, environmental contaminants, or transient species introduced during raw material handling or fermentation processes [36,37]. Through biosequencing technology, the dominant microbiota has been described, which primarily includes bacterial species and fungal strains.

The bacterial community in LTD includes *Lactobacillus*, *Bacillus*, and *Acetobacter*. *Lactobacillus* ferments sugars to produce organic acids such as lactic acid. This process regulates the pH of the fermentation environment and contributes to the sourness and distinctive flavor of *Baijiu*. It also influences the overall mouthfeel of the product [38,39,40]. *Bacillus* species exhibit strong resistance to high temperatures and low-water-activity environments; they contribute to fermentation processes and secrete enzymes such as amylase and protease, which are essential for the saccharification of raw materials and proteolysis [9,41]. *Acetobacter* is an aerobic bacterium that oxidizes sugars and ethanol, contributing to flavor moderation in LFB. However, its excess growth can adversely affect wine quality, necessitating careful control to maintain yeast activity [42,43]. The fungal community in LTD is primarily composed of *Aspergillus*, *Rhizopus*, and *Saccharomyces*. *Aspergillus* produces various enzymes—such as glucoamylase, liquidase, and lipase—that break down macromolecules like starch and cellulose. This process releases sugars and amino acids that support other microorganisms [44,45]. *Rhizopus* exhibits dual functions in both saccharification and fermentation during LFB production. It also produces metabolites, including lactic acid and succinic acid, which contribute to flavor development [46,47]. *Saccharomyces* is the predominant alcohol-producing fungus and plays a critical role in alcoholic fermentation. It generates a wide range of volatile aroma compounds that significantly influence LFB flavor [38,48,49,50]. Although present in limited quantities, *Actinomycetes* also affect the flavor and quality of LFB. These microorganisms can hydrolyze starch, protein, and cellulose, supplying precursors for other microbes. Additionally, they produce flavor components such as ethyl caproate and ethyl butyrate during fermentation [51]. The core microorganisms in LTD collaborate throughout the fermentation process, collectively influencing the flavor and quality of LFB. It is important to note that the stability of this microbial consortium can be influenced by environmental factors such as temperature, which varies seasonally. While the core functional groups remain consistent, their absolute and relative abundances may fluctuate, necessitating careful process control to ensure product consistency. These microorganisms offer a scientific foundation for optimizing brewing processes and enhancing the quality of LFB.

#### 2.1.2. Core Microorganisms Based on Flavor Compounds

The core microbiota of LTD, primarily comprising *Lactobacillus*, *Bacillus*, *Aspergillus*, *Rhizopus*, *Saccharomyces*, and *Candida*, is fundamental to the generation of a diverse spectrum of flavor compounds and precursors through the secretion of essential enzymes [45]. Beyond these dominant genera, low-abundance yet functionally significant microorganisms, including specific *Aspergillus* spp., *Kluyveromyces* spp., and *Saccharomyces cerevisiae*, are critical contributors to the unique flavor profile of LFB [52]. The flavor compound profile is directly shaped by the metabolic activities of this core microbiome. *Aspergillus* species are pivotal producers of enzymes (such as proteases and lipases) that break down proteins and lipids into amino acids and free fatty acids, which are key precursors for thermal reactions during fermentation and aging. They directly generate important flavor compounds such as pyrazines (imparting nutty, roasted notes), various esters (fruity aromas), and aromatic substances [45]. *Kluyveromyces* spp. are recognized as primary producers of ethyl acetate (a key solvent-like aroma) and other short-chain esters that define the aromatic foundation of LFB [53]. *Saccharomyces cerevisiae* not only drives ethanol production but also synthesizes critical terpenoids (contributing floral and citrus notes) and higher alcohols from sugars, profoundly enhancing the flavor complexity [54,55]. Furthermore, lactic acid bacteria (LAB) like *Lactobacillus* and *Weissella* significantly influence flavor by producing organic acids (such as lactic and acetic acid), which acidify the environment and also serve as substrates for esterification reactions, yielding ethyl lactate and other ester compounds [56]. Therefore, by correlating the dynamic changes in microbial community structure with the types and concentrations of flavor compounds (such as esters, pyrazines, acids, and terpenoids) during fermentation, the core functional microorganisms responsible for specific flavor attributes can be precisely identified [52,57,58]. As a vital starter, LTD provides this consortium of microorganisms, enzymes, and pre-formed flavor substances, which directly dictates the final quality and sensory characteristics of LFB [12,14].

#### 2.1.3. Based on Microbial Interactions

In a fermentation system, microorganisms interact closely, ultimately forming a stable community structure. Microorganisms that excel at interaction are recognized as core microorganisms, which play key roles in the stability and functioning of the entire microbial community [59]. Identifying these core microbes is essential in the LTD fermentation process, as it directly influences flavor development and product quality. Traditional fermentation processes feature complex and variable microbial populations, dynamic succession, and interactions, leading to inconsistencies in LTD quality and flavor [60,61]. Therefore, identifying core microorganisms and their interactions not only accelerates the brewing process but also promotes the conversion of flavor compounds, which is critical for enhancing the consistency and stability of LTD quality.

In natural environments, studying microbial interactions helps clarify the structure of microbial communities that work synergistically [62,63,64,65,66]. Microorganisms are not isolated within LTD environments; they exhibit complex relationships, including symbiosis, competition, and antagonism (Table 1), all of which influence community structure and functional stability [10,11,67]. This, in turn, affects LFB fermentation and flavor formation. Understanding microbial interactions is vital for revealing the mechanisms underlying core microbiota formation. In particular, *Bacillus*, as a key genus within the core microbiome, plays a crucial mediating role in shaping community structure through both competitive and antagonistic interactions. For instance, while competing with *Lactobacillus* for nutrients and space, *Bacillus* also exhibits antagonistic effects by producing antimicrobial peptides that inhibit other microorganisms. These dual interactions not only help regulate the population dynamics of *Lactobacillus* but also suppress potential pathogens or opportunists, thereby contributing to the stability and balance of the entire microbial consortium. The interactions among microorganisms are central to enhancing our understanding of the core microbiota in LTD brewing, offering new perspectives on its function and stability. Through co-culture experiments, genomic analysis, and microbial community network analysis, the mechanisms of synergistic interactions can be elucidated [59,68,69,70,71].

In core microbial identification, three methods—namely, those “based on dominant microbial communities,” “based on flavor-related microbial communities,” and “based on microbial interactions”—identify different groups while centering on the core characteristics of “core microbes,” which refers to microbes that play a crucial role in driving LTD fermentation. To clarify the final screening logic for core microorganisms, a multi-dimensional integrated standard must be established. The standard comprises three layers: First, the basic screening layer, which utilizes the “dominant microbial population” method as a quantitative basis, prioritizes the inclusion of microbial communities that exhibit relatively stable and high abundance in LTD, as well as those consistently present across various batches or sources of samples, while excluding communities characterized by occasional high abundance. Second, the functional validation layer combines the “flavor-related microbial communities” method to validate the association between these groups and the characteristic flavors of LTD, such as esters and alcohols, through targeted metabolomics. Even if a group has low abundance, it should still be included in the core library if its metabolic products are signature flavor compounds of LTD. Third, the interaction-essential layer employs the “microbial interaction-based” method to screen for groups that are indispensable in co-culture systems. Although their individual abundance or metabolic contribution may be limited, their absence could lead to the collapse of community function, necessitating their retention as core microorganisms. Ultimately, through a three-dimensional cross-validation process encompassing “abundance stability–functional necessity–irreplaceable interaction,” the core microbial community of LTD has been determined, including species such as *Lactobacillus acetotolerans*, *Bacillus licheniformis*, *Pichia kudriavzevii*, and *Saccharomyces cerevisiae*, all of which must undergo this triple validation [68,77]. Comprehensive multi-dimensional research aids in optimizing LTD quality during fermentation and improving LFB quality [19,78].

### 2.2. Identification of Microbiological Techniques

Traditional microbial identification methods primarily rely on morphological observation and physiological and biochemical characterization. However, these methods have limitations in terms of accuracy and efficiency [21,79]. Consequently, HTS, single-molecule real-time sequencing (SMRT), transcriptomics, metagenomics, metaproteomics, metabolomics, and other molecular biology techniques have been widely adopted in microbial community research [80,81,82]. These advanced identification techniques are particularly prevalent in research on LTD microbes, as they can rapidly and accurately identify microbial species in samples, revealing their community structure and functional genes [82,83,84]. Furthermore, selecting appropriate technological methods (Table 2) can provide new insights for identifying core microorganisms.

#### 2.2.1. HTS and SMRT

HTS methods, such as 16S rRNA and ITS sequencing, are essential for analyzing microbial communities and are commonly used in the field of *Baijiu* research [101,102]. In identifying core microorganisms in LTD, this technology allows for a quick and comprehensive analysis of microbial community composition and diversity [101]. It is primarily used to identify microbial species and their relative abundance by analyzing the variable regions of the 16S rRNA gene to reveal the composition and succession of bacterial biomes, as well as to analyze the ITS region for studying fungal communities [103]. Some studies have employed HTS to analyze the structure of LTD microbial communities, highlighting the diversity and variability among samples from different sources [35]. In LFB brewing, HTS serves multiple roles, including the comprehensive and rapid detection of microbial species and quantities in raw materials, *Daqu*, wine spirits, and pit mud [103,104,105]; tracking microbial succession patterns; investigating assembly and dynamics of microbial communities during the brewing process [106]; exploring the relationship between microbes and flavor; and screening for superior microbial strains suitable for purebred fermentation or process optimization to enhance wine production [107].

SMRT sequencing can identify rare microorganisms and accurately detect those present in extremely low quantities within the LFB brewing environment that may significantly influence flavor formation. For instance, in traditional food fermentations similar to LFB, SMRT and other high-resolution techniques have uncovered the critical roles of low-abundance taxa such as *Lactobacillus*, *Pediococcus*, and specific *yeasts* that contribute to the production of key flavor compounds like esters and organic acids [108,109]. This technique allows for in-depth analysis of microbial species and classification at the single-cell level. Compared to traditional metagenomics and other methods, SMRT can more accurately identify microbial species and subtypes, effectively distinguishing microorganisms with similar morphology or function, thereby enriching our understanding of the LFB microbial community [110]. Moreover, SMRT can analyze specific molecular mechanisms of interspecific interactions, inferring relationships among microorganisms, such as symbiosis, competition, or antagonism, by evaluating gene expression and metabolic characteristics of different microbial cells [47,89]. Additionally, SMRT can track dynamic changes within microbial communities, monitoring alterations at different stages of LFB fermentation and providing insights into the growth, extinction, and functional transformation of microorganisms. For instance, variations in microbial cells can be examined during the early, middle, and late stages of fermentation to determine optimal points for fermentation control [47].

#### 2.2.2. Transcriptomics

Transcriptomics focuses on all microbial transcripts present in environmental and tissue samples at specific times and locations. Utilizing HTS enables the comprehensive acquisition of transcriptional information from both culturable and non-culturable microorganisms. This approach allows researchers to explore the composition of active strains and gene expression in particular environments, as well as analyze the mechanisms through which microbes adapt to environmental stresses and interactions [90,111]. In the field of winemaking, transcriptomics is elucidating the crucial roles of different microorganisms in the fermentation process. Specifically, for the fermentation of Jian-flavor *Baijiu*, research has investigated the “multiple-*qu* fermentation” mechanism through transcriptomics, providing insights into microbial interactions and functions during fermentation [112]. Another study evaluated the effect of ethanol on ester production, examining the transcriptional response of *Wickerhamomyces anomalus* in the presence of lactic acid. This further revealed the mechanisms by which yeasts regulate ester production in alcoholic environments [113].

These findings enhance our understanding of microbial dynamics during fermentation while also establishing a theoretical foundation for optimizing the fermentation process. Transcriptomics comprehensively analyzes gene expression under specific conditions, offering a global perspective. It assists in dissecting cellular metabolism and growth regulation mechanisms, contributing to the identification and understanding of key genes and their functions. Additionally, it focuses on specific biological processes and metabolic pathways, investigates gene expression changes in response to external stresses (such as alcohol and lactic acid), and improves our understanding of microbial adaptation and resilience. Furthermore, transcriptomics reveals how different fermentation processes affect microbial metabolism and flavor synthesis [112,113]. As a powerful tool for studying the transcriptional activities of microbial communities, it provides key insights into microbe–environment interactions and the functionality of microbial communities and can facilitate multidisciplinary research by unveiling the intrinsic mechanisms of complex biological processes at the gene expression level.

#### 2.2.3. Metagenomics

Metagenomic technology can identify microorganisms that traditional culture methods cannot detect. In LTD, numerous low-abundance microorganisms beneficial to *Baijiu* fermentation have also been detected, including *Aspergillus*, *Saccharomyces cerevisiae*, and *Saccharomyces boulardii*. In subsequent LFB brewing processes, these low-abundance microorganisms in LTD warrant further attention [52]. Ref. [96] investigated the core microbial and community composition of two fermenter types (traditional and Round-Koji-mechanical starter) and collected whole-genome data from samples using HTS macrogenomic analysis. This enables species-level analysis and the identification of core differentiating genes associated with carbohydrate, lipid, and amino acid metabolism (based on KEGG and NR databases), thereby identifying core dominant microorganisms.

Several studies have combined metagenomics with metabolite analysis of microbiota, examining the characterization and correlation of higher alcohols, or revealing the mechanism behind n-propanol formation during *Baijiu* fermentation. This research can elucidate the mechanisms of flavor formation in *Baijiu* fermentation and promote the application of biologically innovative technologies in the traditional fermentation industry [114,115]. The application of metagenomic technology in LFB brewing primarily focuses on identifying microbial species, core microorganisms, and screening for superior strains. Furthermore, it facilitates the study of microbial interactions and differences in community composition, the identification of key functional genes, and the analysis of metabolic pathways. It also aids in brewing process optimization, flavor compound research, controlling fermentation processes, and tracing the sources of *Baijiu* quality [95,99,116,117]. Research utilizing this technology provides a scientific basis for optimizing quality and standardizing LFB brewing processes, thereby improving production efficiency and quality stability.

#### 2.2.4. Metaproteomics

Metaproteomics has numerous applications in the field of *Baijiu* [118]. It is employed to analyze the protein composition expressed by microbial communities, linking specific microorganisms to community functions and diversity. The *Daqu* provides saccharification microbiota and enzymes crucial for *Baijiu* brewing, with saccharification enzymes playing a significant role in the fermentation process. Glycolytic enzyme proteins have been detected in two types of aromatic *Baijiu Daqu* [119]. This research identified 77 amylases and cellulases with potential glycolytic abilities, along with significant protein differences in microorganisms such as *Aspergillus* spp. and *Rhizoctonia* spp., highlighting key proteins and functional sources in *Daqu*. This work provides a foundation for optimizing the production process of *Daqu*. Other studies have focused on optimizing microbial structure and function by modulating exogenous factors to enhance the quality of saccharifiers and the aromatic characteristics of fermented foods and beverages [120]. Taking LFB as an example, HTS and label-free quantitative proteomics were employed to identify key enzymes and microorganisms involved in saccharification and fermentation. Metaproteomics analysis revealed that *Lichtheimia* is a significant producer of α-amylase and glucosidase during LTD fermentation and also contributes to the formation of flavor compounds, thereby benefiting the overall fermentation process [14].

Metaproteomics, combined with other multi-omics techniques in microbial community structure analysis, can identify microorganism types and quantities, clarify their distribution, and analyze community structure differences. In optimizing the brewing process, it can guide LTD production and monitor fermentation, while also studying flavor formation mechanisms by revealing the metabolic pathways of flavor substances and identifying key flavor enzymes and microorganisms. When examining the fermentation mechanism, metabolic pathway analysis can explore the regulatory roles of functional proteins; in the identification and analysis of functional proteins, key enzymes can be studied and differential proteins analyzed [82,84,121,122]. This technology is crucial for quality control and standardization, as it helps discover quality markers and facilitates the standardization of LFB production.

#### 2.2.5. Metabolomics

Metabolomics is a technique for comprehensively analyzing an organism’s metabolites [123]. Through metabolomics analysis, key metabolites related to *Baijiu* flavor in *Daqu* and their producing strains have been identified, revealing the metabolic pathways and products of the core microorganisms in fermentation. In investigating the flavor formation mechanisms in *Baijiu*, techniques such as gas chromatography–mass spectrometry (GC-MS) and liquid chromatography–mass spectrometry (LC-MS) can identify key flavor metabolites, including esters and alcohols, while also unveiling the metabolic pathways of flavor-producing microorganisms, such as lactic acid from *Lactobacillus* and *Saccharomyces*, and the role of ethanol in flavor formation [64]. Ref. [56] employed shotgun metagenomics combined with metabolomics to study the LFB fermentation microbiome, analyze its structure and characteristic metabolites, establish the relationships between microorganisms and metabolites, and clarify the core microbial metabolic potential. The findings indicated that *Lichtheimia ramose*, *Saccharomycopsis fibuligera*, and *Bacillus licheniformis* were the primary contributors to starch saccharification, while *Saccharomyces cerevisiae* and *Pichia kudriavzevii* were responsible for ethanol production. Different kinds of *Lactobacillus* species contributed to fermentation and acted as the main flavor-producing microorganisms during the late stage of fermentation. When studying microbial community structure and function, metabolomics combined with HTS can analyze community composition and explore microbial interactions [124,125]. In monitoring the LTD fermentation process and quality control, metabolomics can conduct real-time process monitoring and timely detection of issues; the unique metabolomic profiles of LTD from different origins and processes serve as a basis for quality assessment and traceability, establishing fingerprints to identify LFB quality and prevent the production of inferior products [126].

The mechanisms of prokaryotic classification and functional dynamics in Artificial Pit Mud (APM) and its relationship with high-quality *Baijiu* production have been investigated [82]. In that study, the brewing microcosm was analyzed for the first time using a combination of macrogenomics, metaproteomics, and metabolomics. A total of 36 prokaryotic classes and 195 genera were detected, with *Bacilli* and *Clostridia* being dominant; notably, the relative abundance of *Bacilli* decreased with APM maturation. An integrated analysis of multi-omics data can reveal the complex relationships between gene regulation and metabolic pathways. Utilizing multi-omics technologies not only provides a more comprehensive understanding of biological processes but also identifies new biomarkers and potential directions for industrial applications. A comparison of various microbial identification technologies indicates that HTS rapidly and efficiently analyzes microbial community composition and diversity, making it suitable for large-scale sample analyses while revealing dynamic changes in microorganisms throughout different fermentation stages. Meanwhile, SMRT can deeply analyze individual microbial genomic information, uncovering community heterogeneity and functional distinctions that traditional sequencing may mask. Transcriptomics can analyze tens of thousands of genes simultaneously, providing comprehensive gene expression information and differential expression analysis. It identifies significantly differentially expressed genes by comparing gene expression across different samples or treatment conditions, utilizing KEGG and other databases to conduct functional annotation and metabolic pathway enrichment analyses, thereby elucidating the roles of these genes in biological processes and ensuring the accuracy and reproducibility of analysis results [112,113,127,128].

Metabolomics explores the potential functions of microorganisms at the genetic level, identifying key functional genes and providing insight into the gene pool of microbial communities. Metabolomics directly reveals the protein expression of microorganisms, verifying metabolic activity and reflecting their functional status in real environments [129]. Metabolomics uncovers the microbial metabolic networks and their influence on LFB flavor by analyzing fermentation products, providing a basis for optimizing brewing processes. HTS, SMRT, transcriptomics, metagenomics, metaproteomics, and metabolomics collectively enable multi-level analysis of microbial diversity, functions, and metabolic pathways during LFB fermentation, encompassing genes, transcripts, proteins, and metabolites. By revealing dynamic changes and functional differences in microbial communities, these technologies provide a scientific basis for optimizing brewing processes and enhancing the quality and flavor stability of LFB, playing a crucial role in studying LFB microbial community structures and laying a theoretical foundation for developing new LFB varieties. In the future, integrating and analyzing multi-omics data will become a significant direction for research, with the potential to realize personalized customization in LFB brewing, advancing the industry’s modernization and competitiveness and demonstrating considerable promise in LFB microbiology research.

## 3. SynComs

The construction of SynComs should be based on identified LTD core microorganisms, establishing a complete chain of “identification–screening–assembly–verification.”

### 3.1. Concepts

SynComs are artificially designed and constructed microbial systems composed of various wild-type bacterial species or engineered strains, combined in specific ratios to serve defined functions. In the domain of LFB brewing, these systems can replicate the LTD microbial community, optimize the fermentation process, and enhance the quality and flavor of *Baijiu*.

### 3.2. Advantages and Applications

SynComs are microbial communities with targeted functions, formed through the artificial screening, combination, and design of multiple microorganisms. Recently, they have garnered significant attention in industrial production and environmental fields [24,25,130,131,132]. Natural microbial systems rarely consist of isolated species; extensive ecological evidence indicates a positive correlation between species diversity and the functional output of communities. These communities are prevalent in nature, suggesting potential benefits of engineered strains in co-culture models [133,134,135,136,137].

The application of SynComs in *Baijiu* fermentation has garnered significant attention from researchers. It has been established that *Pichia*, predominantly of environmental origin, is the primary fungal genus throughout the stacking fermentation process and that it can contribute to the establishment of the *Baijiu* fermentation microbial community. This underscores the crucial role of environmental microbiota in shaping and facilitating the development of this community [138]. Moreover, research indicates that increasing the ethyl caproate synthesis capacity of microorganisms with high esterase activity enhances the fermentation process of *Zaopei.* Biofortification has proven to be an effective strategy for improving the quality of the stock solution and augmenting the ethyl caproate content [139]. In LFB brewing, SynComs can significantly enhance fermentation efficiency, reduce fermentation cycles, and lower production costs by optimizing microbial composition and ratios. The application of SynComs can also facilitate standardized production and improve product stability and consistency [43,67,83,140]. Under LTD fermentation conditions, the activity of natural microbial communities is often inhibited, resulting in slow fermentation initiation and insufficient aroma production. SynComs introduce functionally robust strains adapted to low temperatures, such as cold-tolerant *Lactobacillus plantarum*, *Weissella cibaria*, and *Leuconostoc mesenteroides*, facilitating the rapid establishment of dominant communities at 10–15 °C. This significantly shortens fermentation cycles and enhances fermentation stability [27]. In LFB fermentation, the application of SynComs has been shown to effectively enhance the synthesis of key flavor compounds. To optimize the metabolism of guaiacol, ref. [141] demonstrated that a three-strain SynCom comprising *Wickerhamomyces anomalus* and two cold-adapted bacteria (*Pantoea* sp. and *Bacillus altitudinis*) enables the synergistic metabolism of ferulic acid at low temperatures, promoting the efficient conversion of 4-vinyl guaiacol to 4-ethyl guaiacol, thereby enhancing the fruity and floral characteristics of *Baijiu*. Additionally, SynComs can increase the accumulation of medium-chain fatty acids and their ethyl ester derivatives under low-temperature conditions by regulating fatty acid metabolic pathways, including OLE1, FAA1, and other gene expressions. This further improves the balance between ester aroma and taste in LFB [142]. Compared with traditional natural inoculation [27,141], SynComs exhibit greater stress resistance and metabolic consistency during low-temperature fermentation, reducing batch-to-batch variations caused by environmental fluctuations and offering a new approach for LFB to achieve standardized and controllable production.

SynComs allow for precise control over microbial composition, enabling more stable execution of specific fermentation functions while minimizing interference from environmental factors compared to natural communities. Furthermore, the synergistic effects among microorganisms can be enhanced through intentional design, which improves fermentation efficiency and yield of target products. These advantages position SynComs as exemplary not only in food fermentation but also in biotechnology processing, environmental treatment, and various other applications, presenting a wide range of potential prospects for providing innovative ideas and methods for related industries. Constructing SynComs addresses the frequent issues encountered in engineered monocultures, particularly as functional complexities increase [143]. Compared to traditional natural fermentation, SynComs provide improved controllability, stability, and efficiency while reducing complexity, thereby significantly optimizing the fermentation process, particularly in food fermentation, and demonstrating substantial advantages [24].

### 3.3. Shortcomings and Challenges

Although SynComs have demonstrated significant benefits in food applications, particularly in LTD-related applications, their widespread promotion and in-depth application face numerous complex challenges.

During the community construction and design phase, it is challenging to screen microorganisms that are well adapted to low-temperature environments. LTD fermentation typically occurs at temperatures between 45 and 50 °C, necessitating that participating microorganisms possess good low-temperature adaptability [1]. Precisely identifying a combination of microorganisms from a diverse pool of environmental microorganisms that can function efficiently at low temperatures and effectively promote saccharification, fermentation, and flavor formation during the LTD fermentation process is a complex task. Moreover, determining the optimal ratio of these microorganisms in SynComs is notably challenging. Growth rates and metabolic activities of different microorganisms can vary significantly in low-temperature environments; even a slight imbalance in their ratios may hinder the effective utilization of community functions.

Maintaining community stability presents another major challenge. LTD fermentation cycles are relatively lengthy, and during this time, even minor fluctuations in environmental factors such as humidity and oxygen content can adversely affect the microbial composition and functionality of SynComs [1]. Maintaining community stability presents another major challenge. LTD fermentation cycles are relatively lengthy, and during this time even minor fluctuations in environmental factors such as humidity and oxygen content can adversely affect the microbial composition and functionality of SynComs [52]. Furthermore, ensuring consistent fermentation conditions in large-scale production is challenging, as variations among batches can exacerbate issues in maintaining SynCom stability and compromise product quality consistency. Currently, there are no comprehensive and targeted standards to regulate the research, development, production, and application of SynComs in LFB production. It is essential to conduct thorough assessments of the long-term impacts of introducing SynComs on the safety, flavor characteristics, and consumer health of LFB products. For instance, further research is required to determine whether newly constructed SynComs produce unknown metabolic byproducts that may pose potential risks to human health [144,145].

Additionally, current research techniques impose limitations on the in-depth exploration of SynCom applications in LTD. Existing HTS and other technologies are insufficient for real-time, high-precision monitoring of dynamic changes in microbial communities during the LTD fermentation process in low-temperature environments [144]. This limitation hinders the acquisition of critical information regarding microbial interactions within the community and metabolic pathway changes, which can obstruct timely optimizations of SynComs’ construction and application strategies. Therefore, to fully harness the potential of SynComs in LTD fermentation, it is imperative to conduct in-depth research and foster collaborative efforts across multiple domains, including microbial screening, maintenance of community stability, and technological innovation. While Synthetic Microbial Communities show promising potential for improving the reproducibility and quality of *Baijiu* fermentation, most studies to date have been conducted at laboratory scale. There is currently limited published data on the performance of such communities in pilot or commercial-scale processes. Challenges related to scaling up, including environmental variability, microbial dynamics, and process control, remain to be thoroughly addressed. Future work focusing on translational validation will be essential for bridging this gap.

### 3.4. Construction Methodology

Core microbiome mining is founded on the “bottom-up design” concept, which aims to identify and characterize the common interaction patterns and mechanisms among bacterial species by screening, combining, and optimizing microbial communities. These patterns and mechanisms constitute key drivers of overall community structure and dynamic changes, facilitating the construction of functionally stable artificial SynComs [146,147,148].

Screening represents the initial step in the construction of SynComs and typically focuses on target functions. In studies of LTD microbial communities, screening identifies prevalent and dominant microbiota, microorganisms critical for fermentation and flavor formation, and symbiotic microbiota [60,149]. Specific methods include (1) identifying dominant microorganisms in LTD through biotechnological means and (2) isolating strains with specific functions, such as aroma or enzyme production, from LTD. These identified core microorganisms form the foundation for SynCom construction [78,150].

During the assembly phase, selected core microorganisms are mixed in specified ratios to create the initial SynComs. This process must consider microbial interactions, synergistic effects, and environmental influences [151]. Co-culture experiments and fermentation performance tests are conducted to screen for strain combinations that demonstrate synergistic functionality [152].

Optimization is a crucial element in building SynComs and comprises the following steps: (1) evaluating the fermentation performance and flavor production capacity of the cluster through laboratory fermentation experiments to identify the optimal combination; (2) utilizing systems biology and computer simulations to predict microbial interactions and community dynamics to inform community construction; (3) applying gene editing techniques, such as the CRISPR-Cas system, for precise modifications of microbial genes to enhance specific functions [153]; (4) employing biomics technology for the precise regulation of microbial functions to establish microbial communities with targeted metabolic pathways; and (5) optimizing community structure and environmental conditions based on microbial ecology principles to ensure stability and efficient operation.

**Figure 2:**
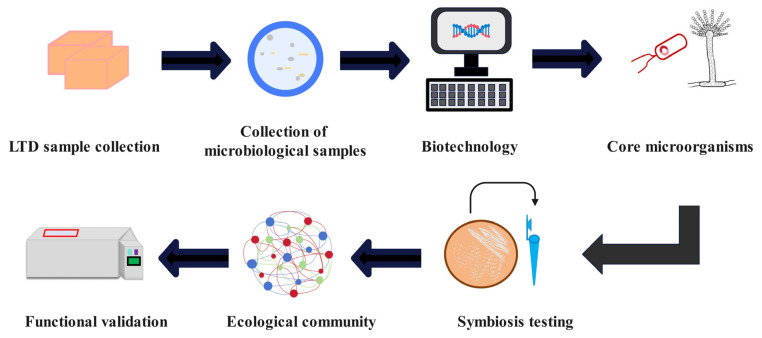
SynComs build steps for LTD.

Utilizing optimized SynComs in fermentation represents an effective method to regulate and control flavor metabolism in traditional fermented foods. Taking LFB as an example, this method encompasses the following steps (Figure 2): (1) LTD sample collection, involving the gathering of microbiological samples; (2) biotechnological identification of dominant microorganisms; (3) identifying the core microbiome through the mining of core functional microorganisms via symbiotic network analysis and functional predictions based on biotechnology data [149,154]; (4) screening, involving targeted isolation and culture of core microbial strains; (5) symbiosis testing, which includes functional optimization and assessments of symbiosis and antagonism among core strains to evaluate performance; (6) colony construction by mixing the best-performing microorganisms in specific ratios; and (7) functional validation, which assesses metabolic activity and other functional indicators of synthetic communities under natural or controlled conditions [24,141,155].

Core microbiome mining and SynCom construction constitute a systematic project involving screening, combination, optimization, and functional verification. By integrating multidisciplinary technologies, SynComs with stable functions and superior performance can be developed, providing a scientific foundation for process optimization and quality enhancement in LFB fermentation and other domains. Identifying core microbiota and constructing SynComs within ecosystems have become widely used methods for elucidating the performance and stability of microbial communities. With advancements in synthetic biology and systems biology, this field is poised for broader applications in the future.

## 4. Summarizing the Outlook

The synthesis of LTD colonies has emerged as a prominent area of focus within LFB fermentation and biotechnology, emphasizing the composition, function, and relationship of LTD microorganisms with wine flavor and quality. Recent years have witnessed significant advancements in the identification of dominant core groups, including *Lactobacillus*, *Bacillus*, *Acetobacter*, *Aspergillus*, *Rhizopus*, and *Saccharomyces*, and in the construction of SynComs, aided by multi-omics technology. However, the field continues to encounter challenges, such as a lack of understanding regarding the ecological functions and regulatory mechanisms of LTD microbial communities, as well as the need for improved stability and adaptability of SynComs. Future research directions should prioritize an in-depth exploration of the relationship between microorganisms and LFB quality, optimization of microbial modification processes, construction of tailored microbial communities for various types of *Baijiu*, and investigation of the stability and adaptability of SynComs. As microbial research in LFB fermentation advances, the ongoing development of biotechnology promises to yield innovations that will support the sustainable growth of the LFB industry.

## Figures and Tables

**Table 1:** Core microbial interactions in LTD.

Type of Interaction	Typical Combinations	Functional Performance	References
Symbiosis	*Rhizopus* and *Saccharomyces*	Saccharification–fermentation synergy; *Rhizopus* has a strong amylase production capacity, which can directly degrade the starch in raw materials into reducing sugars that can be utilized by *Saccharomyces*, thus stimulating the growth of *Saccharomyces*, and the two synergistically promote the smooth progress of the fermentation process	[72,73]
	*Lactobacillus* and *Saccharomyces*	*Lactobacillus* metabolizes organic acids to lower the environmental pH and inhibit the growth of harmful microorganisms, while at the same time providing a suitable environment for *Saccharomyces*, which produces ethanol and carbon dioxide through fermentation, providing carbon and energy for *Lactobacillus*	[68,73,74]
Competition	*Lactobacillus* and *Bacillus*	*Lactobacillus* utilizes the raw materials to produce organic acids such as lactic acid during the fermentation of macrocystis, which leads to a lowering of the pH of macrocystis, thus inhibiting the growth of *Bacillus*, which competes for nutrients and space to live with *Lactobacillus*, and the two compete with each other, affecting the growth and metabolism of each other	[75]
	*Lactobacillus* and *Acetobacter*	In the context of pH regulation during LTD fermentation, *Lactobacillus* and *Acetobacter* compete for sugars and other nutrients. When *Lactobacillus* dominates, it produces lactic acid, which reduces the pH, inhibits spoilage bacteria, and contributes to aroma through the formation of ethyl lactate. In contrast, dominance by *Acetobacter* leads to the production of acetic acid and ethyl acetate, which may adversely affect wine quality	[9]
Antagonism	*Bacillus* produces antimicrobial peptides to inhibit bacteria	*Bacillus* exerts an inhibitory effect on other microorganisms, and in LTD fermentations this inhibitory effect is usually controlled to maintain the balance of the microbial community	[76]

**Table 2:** Strengths and weaknesses of microbial identification techniques.

Identification of Microbiological Techniques	Strengths	Weaknesses	References
HTS	Comprehensive detection; high sensitivity; high resolution and efficiency; multidisciplinary application	Complexity of data processing and analysis; high cost of technology; difficulty in validating microbial functions; technical limitations	[85,86,87]
SMRT	Long reads spanning repeated sequences; complete gene assembly; real-time monitoring of base modifications; capturing transient changes; high accuracy and flexibility	Low throughput; limited sample coverage; high cost; complex data analysis and processing	[88,89]
Transcriptomics	Visualizes the actual functional status of microbial communities; captures gene expression in rapid response; applicable to a wide range of sample types and treatment conditions; compares differentially expressed genes and functional pathways in different environments to reveal microbial adaptive strategies	Different bacterial sequences in microbiome samples are intertwined with each other, making it difficult to accurately distinguish the species origin of homologous sequences; it is difficult to analyze intercellular heterogeneity; it is difficult to extract RNA, and mRNA is unstable and out of sync with protein expression, so it needs to be integrated with multiple histologies; the amount of data is large, and the analysis requirements are high	[90,91,92,93,94]
Metagenomics	Direct DNA sequencing of environmental samples without culturing microorganisms; comprehensively analyzes microbial communities; analyzes species composition, functional genes, and metabolic potential; reveals diversity and function; explores functional genes and discovers genes with potential application value; studies microbial interactions and their relationship with the environment; applicable to a wide range of environmental samples	Complexity of data analysis; limited species resolution; difficult to distinguish highly similar species or strains; incomplete functional annotations; limitations in functional prediction; easy to introduce bias in sample processing, affecting the accuracy of the results; high cost, limiting large-scale studies	[45,67,95,96,97]
Metaproteomics	Directly reflects functional information; breaks through uncultured microbial limitations; reveals microbe–host interactions; integrates multi-omics data; high sensitivity and resolution	Complex sample preparation; difficult data analysis; difficult quantitative analysis; large variation in protein abundance; poor database	[32,45,98]
Metabolomics	Comprehensive detection; comprehensive analysis of metabolites; complementary with other histologies, revealing regulatory mechanisms; easy to detect; wide range of applications; high sensitivity; diverse technical means	Difficulty in detection and quantification; low-abundance metabolites are difficult to detect; complexity of data analysis, lack of uniform standards; difficulty in metabolite identification; high research costs	[36,56,99,100]

## Data Availability

No new data were created or analyzed in this study.

## Abbreviations

The following abbreviations are used in this manuscript:

LFB	Light-flavor *Baijiu*
LTD	Low-temperature *Daqu*
SynComs	Synthetic Microbial Communities
PCR	Polymerase chain reaction
HTS	High-throughput sequencing
SMRT	Single-molecule real-time sequencing
GC-MS	Gas chromatography–mass spectrometry
LC-MS	Liquid chromatography–mass spectrometry
APM	Artificial Pit Mud
